# Respiratory Syncytial Virus Infection, TLR3 Ligands, and Proinflammatory Cytokines Induce CD161 Ligand LLT1 Expression on the Respiratory Epithelium

**DOI:** 10.1128/JVI.02789-13

**Published:** 2014-03

**Authors:** Stifani Satkunanathan, Naveenta Kumar, Monika Bajorek, Marco A. Purbhoo, Fiona J. Culley

**Affiliations:** a Department of Respiratory Medicine, Centre for Respiratory Infection, MRC and Asthma UK Centre in Allergic Mechanisms of Asthma, National Heart and Lung Institute, Faculty of Medicine, Imperial College London, London, United Kingdom; b Section of Gastroenterology and Hepatology, Department of Medicine, Faculty of Medicine, Imperial College London, London, United Kingdom; c Section of Virology, Department of Medicine, Faculty of Medicine, Imperial College London, London, United Kingdom

## Abstract

During respiratory-virus infection, excessive lymphocyte activation can cause pathology both in acute infection and in exacerbations of chronic respiratory diseases. The costimulatory molecule CD161 is expressed on lymphocyte subsets implicated in promoting respiratory inflammation, including Th2, Th17, mucosally associated invariant T (MAIT) cells, and type 2 innate lymphoid cells. We asked whether the CD161 ligand LLT1 could be expressed on respiratory epithelial cells following respiratory-virus infection as a mechanism by which respiratory-virus infection could promote activation of proinflammatory lymphocytes. In response to respiratory syncytial virus (RSV) infection, expression of LLT1 was upregulated in the BEAS-2B respiratory epithelial cell line and primary human bronchial epithelial cells. Imaging studies revealed that LLT1 expression increased in both RSV-infected and cocultured uninfected cells, suggesting that soluble factors produced during infection stimulate LLT1 expression. TLR3 and TLR2/6 ligands led to a rapid increase in LLT1 mRNA in respiratory epithelial cells, as did the proinflammatory cytokines type I interferons, interleukin 1β (IL-1β), and tumor necrosis factor alpha (TNF-α), which are produced early in respiratory-virus infection. Immunohistochemistry confirmed the increase in LLT1 protein on the epithelial cell surface, and live-cell confocal microscopy demonstrated accumulation of epithelial LLT1 at synapses formed with CD161^+^ T lymphocytes. LLT1 expression by the respiratory epithelium in response to respiratory-virus infection and inflammatory cytokines represents a novel link between innate immunity and lymphocyte activation. As a regulator of CD161^+^ proinflammatory lymphocytes, LLT1 could be a novel therapeutic target in inflammation caused by respiratory-virus infection.

**IMPORTANCE** The immune response to respiratory-virus infection is essential for clearing the pathogen but, if excessive, can lead to tissue damage and obstruction of the airways. How viral infection activates immune cells in the lungs is not fully understood. Here, we show that LLT1 can be expressed in lung cells in response to infection. LLT1 triggers CD161, a receptor on inflammatory immune cells. This mechanism may promote activation of immune cells in the lungs in viral infection and could be a novel target for therapies aimed at reducing lung inflammation.

## INTRODUCTION

In acute respiratory-virus infection, such as bronchiolitis caused by respiratory syncytial virus (RSV) infection, excessive lymphocyte activation can cause airway damage and occlusion, leading to loss of vital gaseous exchange. Furthermore, in chronic diseases, such as asthma, inflammation is exacerbated by respiratory-virus infection, which promotes inflammation driven by lymphocytes, particularly Th2 and Th17 cells (1–3). Understanding how lymphocyte activity in the lungs is exacerbated by respiratory-virus infection is therefore an important goal.

CD161 is a costimulatory molecule found on proinflammatory lymphocytes in the lungs and was first identified as a natural killer (NK) cell receptor (4). Ligation of CD161 is inhibitory for NK cell function but can promote T cell activation, proliferation, and cytokine secretion (5–10). CD161 is expressed on a large proportion of T cells at mucosal surfaces and on CXCR6^+^ cells that traffic to the lung (11–14). All Th17 lymphocytes express CD161 (11, 15–17), and CD161^+^ Th17 lymphocytes have been implicated in promoting inflammation in RSV bronchiolitis, in allergic pulmonary inflammation, and in asthma exacerbation (18–22). CD161 can also be expressed on CD8^+^ T cells, γδ T cells, NK T cells, and other innate lymphoid cells that can promote inflammation in the airways (13, 23). Interestingly, recent data suggest that cross-linking of CD161 on mucosally associated invariant T (MAIT) cells, which are highly CD161-expressing lymphocytes abundant at mucosal surfaces, may modulate cytokine responses, but not cytotoxicity (24).

As CD161 is expressed on proinflammatory lymphocytes implicated in driving respiratory inflammation, we wished to understand how CD161 signaling is controlled in the lung. We asked whether the CD161 ligand lectin-like transcript 1 (LLT1) was expressed on respiratory epithelial cells during respiratory-virus infection (7, 25). LLT1 expression has been shown on peripheral-blood-derived leukocytes, but its expression in the lung has not been studied (8, 10). Here, we demonstrate that LLT1 expression is very rapidly upregulated on the surfaces of bronchial epithelial cells in response to respiratory syncytial virus infection. Furthermore, proinflammatory cytokines released during respiratory infection and stimulation of Toll-like receptors (TLR), including TLR3, could also mediate LLT1 expression. Stimulation of LLT1 transcription leads to cell surface expression of LLT1 protein, which clusters at the immunological synapse with CD161-expressing T lymphocytes. Thus, the CD161-LLT1 axis provides a molecular link between respiratory-virus infection and regulation of inflammatory lymphocytes in the lung and is a potential novel therapeutic target in respiratory inflammation.

## MATERIALS AND METHODS

### Cell culture.

The transformed human bronchial epithelial cell line BEAS-2B (ATCC and LGC Standards, United Kingdom) was cultured in RPMI 1640 medium supplemented with penicillin (100 U/ml), streptomycin (100 μg/ml), l-glutamine (0.8 mM) (culture medium), and 10% heat-inactivated fetal calf serum (FCS) (all from PAA Laboratories). Primary isolates of normal human bronchial epithelial (NHBE) cells (Lonza) were cultured using Clonetics BEBM medium supplemented according to the manufacturer's instructions with bovine pituitary extract, insulin, hydrocortisone, gentamicin, amphotericin sulfate, transferrin, triiodothyronine (T_3_), adrenaline, human epidermal growth factor, and retinoic acid.

### Respiratory syncytial virus.

Plaque-purified RSV strain A2 (ATCC) was propagated in Hep-2 cells. The viral titer was determined by infection of Hep-2 cell monolayers for 1 h in serum-free medium, followed by addition of culture medium containing 4% FCS. The titer was confirmed on BEAS-2B monolayers under the conditions used for experiments. To produce UV-inactivated virus, RSV stock was irradiated with UV light for 1 min using a UVP CX-2000 UV cross-linker (Ultra-Violet Products Ltd., United Kingdom), and inactivation was confirmed by plaque assay.

### Experimental stimulations and infections.

Six-well culture plates were coated in basement membrane matrix (Geltrex; Invitrogen) diluted to 0.1 mg/ml in RPMI. BEAS-2B cells (0.5 × 10^6^ per well) in culture medium containing 2% FCS were added and allowed to form an adherent monolayer overnight. For viral infections, RSV was added at a multiplicity of infection (MOI) of 1.0 in serum-free RPMI medium for 2 h, and then, an equal volume of 4% FCS culture medium was added to each well and the plates were incubated for the desired length of time.

The following cytokines and TLR agonists were used for stimulations: interleukin 1β (IL-1β), IL-10, IL-17, IL-22, gamma interferon (IFN-γ), transforming growth factor beta (TGF-β), IFN-λ1, IFN-λ2, IFN-α, IFN-β, and IFN-ω (all from Peprotech, United Kingdom) and the TLR agonists lipopolysaccharide (LPS), Pam3CSK4, HKLM, poly(I·C) high molecular weight (HMW), poly(A·U), imiquimod, R848, CL075, single-stranded RNA 40 (ssRNA40)/Lyovec, ssRNA41/Lyovec, and ODN 2006 (all from Invivogen). All stimulations were carried out in culture medium with 2% FCS.

### RT-PCR analysis of LLT1 expression.

Total RNA was extracted from cells using TRIzoL reagent (Invitrogen), the quantity and purity were determined using a NanoDrop 1000 spectrophotometer (Thermo-Fisher Scientific Inc.), and RNA was reverse-transcribed into cDNA using a high-capacity RNA-to-cDNA kit (Invitrogen) according to the manufacturer's instructions. A real-time reverse-transcription (RT)-PCR assay for LLT1 expression (*clec2d* gene) was performed using a TaqMan gene expression assay (Applied Biosystems), and the transcript levels were expressed relative to a GAPDH (glyceraldehyde-3-phosphate dehydrogenase) endogenous control (Applied Biosystems). The fold change in LLT1 expression was calculated relative to unstimulated cells following normalization to GAPDH expression and was expressed as 2^−ΔΔ*CT*^ (Δ*C_T_* is the difference in threshold cycles for the test gene and GAPDH; ΔΔ*C_T_* is the difference in Δ*C_T_* between stimulated cells and unstimulated controls).

### Confocal imaging of RSV and LLT1.

Overnight cultures of BEAS2B cells, seeded at 4 × 10^5^ cells per well in 6-well plates containing 16-mm coverslips, were infected with RSV at an MOI of 1.0, and 20 h after infection, the cells were fixed with 4% paraformaldehyde, blocked with 3% bovine serum albumin (BSA) in 0.2% Triton X-100, phosphate-buffered saline (PBS) for 10 min, and coimmunostained with polyclonal anti-RSV (1:200; Abcam) and anti-LLT1 (1:200; Novus Biologicals) for 1.5 h, followed by secondary antibodies, anti-goat conjugated to Alexa Fluor 488 (AF488) (RSV) and anti-mouse AF568 (LLT1) (1:1,000; Invitrogen), for 45 min. Images were obtained on a Zeiss 5 Pascal confocal laser scanning microscope using a 63×/1.4 Plan-Apochromat oil lens (averaging 4 times). Images were acquired using Zeiss LSM Image Browser software (4.2.0.121; Zeiss).

### Immunohistochemistry.

BEAS-2B cells were plated at a density of 2.5 × 10^4^ per well in an 8-well chamber slide (Nunc, Rochester, NY), stimulated as described above for 16 h, and fixed with 10% formalin. The wells were blocked and then incubated with primary mouse LLT1 monoclonal antibody (MAb) (Novus Biologicals) or mouse IgG isotype control at 4°C overnight, followed by rat anti-mouse IgG1 secondary antibody (BD) and avidin-peroxidase (Sigma), and were developed with 3,3′-diaminobenzidine (DAB) substrate.

### T cell transfection and flow cytometry.

Jurkat leukemic T cells were transiently transfected with CD161-enhanced yellow fluorescent protein (EYFP) cloned into mammalian expression plasmid pcDNA3.1 (Invitrogen) by microporation (Digital Bio Technology, Seoul, South Korea), with a single pulse of 30 ms at 1,380 V. After 24 h, the cells were stained with LIVE-DEAD aqua stain (Invitrogen), followed by anti-CD161-allophycocyanin (APC) (clone 191B8; Miltenyi Biotech). Flow cytometry was performed on a BD Fortessa instrument, and the data were analyzed using FloJo software (Tree Star, Inc.), gating for live singlet cells before analysis of CD161-EYFP and surface CD161 expression, detected with the CD161 antibody.

### Live-cell confocal imaging.

BEAS-2B cells were transfected with LLT1-EYFP cloned into mammalian expression plasmid pcDNA3.1 (Invitrogen) using jetPrime reagent (Polyplus Transfection), following the manufacturer's protocol, and 0.5 μg of plasmid.Stable transfectants were selected using G418. LLT1-EYFP expressing BEAS-2B cells were seeded onto treated chambered coverslides (Nunc, Rochester, NY) and left overnight to adhere. Jurkat leukemic T cells were transfected with CD161-mCherry as described above. The transfected Jurkat T cells were dropped onto BEAS-2B cells, and the cellular interactions were imaged by resonance scanning confocal microscopy using laser lines of 514 and 594 nm and a 63× 1.2-numerical-aperture (NA) water immersion objective (TCS SP5 RS; Leica, Heidelberg, Germany). Live-cell images were developed at 37°C and 5% CO_2_, using 10% FCS-RPMI as the imaging medium. Images were developed using Leica Application Suite Advanced Fluorescence (Leica, Heidelberg, Germany) software. Simultaneous imaging of different fluorophores was done by sequential line scanning (TCS SP5).

### Data analysis.

The fold change in expression of mRNA was quantified from RT-PCR data by the 2^−ΔΔ*CT*^ method using Microsoft Excel before importing into GraphPad Prism (GraphPad Software, USA) for production of graphs and statistical analysis. For multiple comparisons of test samples to controls, one-way analysis of variance (ANOVA) was used with Dunnett's posttest, and *P* values of <0.05 were considered significant.

## RESULTS

### LLT1 is expressed on respiratory epithelial cells in response to RSV infection and is dependent on replicating virus.

**FIG 1:**
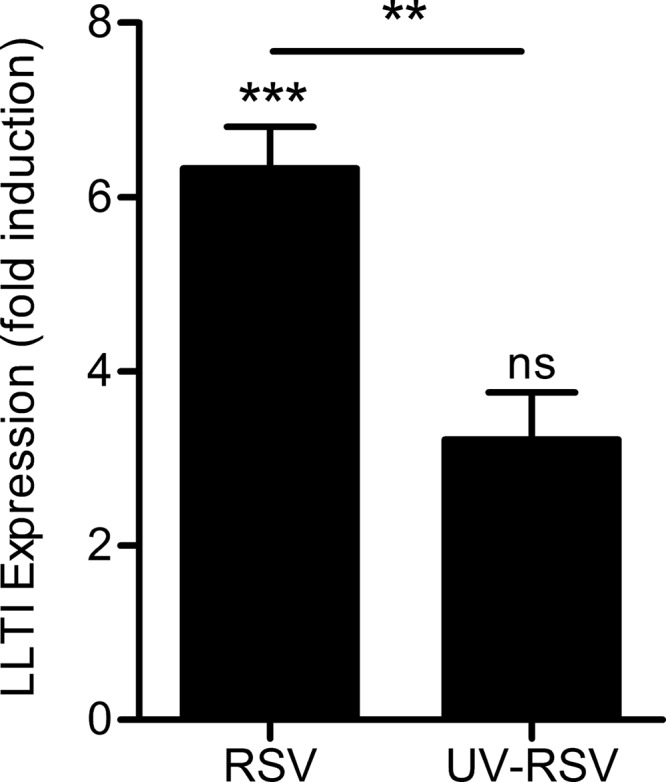
LLT1 is expressed in response to respiratory syncytial virus infection. BEAS-2B cells were infected with respiratory syncytial virus at an MOI of 1.0, and expression of LLT1 was analyzed 6 h later by quantitative PCR. The data are shown as fold induction above unstimulated control cells, normalized to the GAPDH housekeeping gene. The data are means and standard deviations (SD) of triplicate wells. ns, not significant; ***, *P* < 0.001, and **, *P* < 0.01 by one-way ANOVA in comparison to unstimulated cells; the bar indicates comparison between groups.

Respiratory-virus infection can cause acute inflammation and exacerbation of existing chronic inflammatory diseases, such as asthma. We wished to determine whether infection of the epithelium with RSV could stimulate LLT1 expression. BEAS-2B cells were infected at an MOI of 1.0 for 1 h in serum-free medium, followed by addition of medium containing serum for a further 5 h. We found basal expression of LLT1 in the epithelial cells and that LLT1 expression was further induced by infection. We wanted to determine if rapid induction of LLT1 by RSV infection was due to the presence of replicating virus or recognition of virus particles or due to another component of the RSV stock, which is a relatively crude preparation made from infected Hep-2 cells. RSV was UV inactivated for 1 min, and inactivation was confirmed by plaque assay. LLT1 expression was greatly reduced following UV inactivation of RSV (Fig. 1), which reveals that LLT1 expression is promoted by replicating virus.

### LLT1 is expressed in RSV-infected and cocultured uninfected cells.

**FIG 2:**
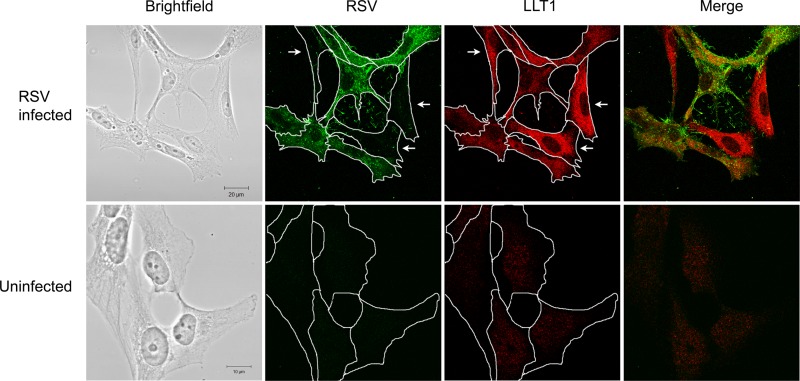
LLT1 is expressed in cocultured RSV-infected and uninfected respiratory epithelial cells. BEAS2B cells were infected with RSV at an MOI of 1.0. After 20 h, the cells were stained with anti-RSV, followed by an AF488-conjugated secondary antibody (green) to identify RSV-infected cells and anti-LLT1 followed by an AF568-conjugated secondary antibody to stain for LLT1 protein (red). Representative images show that both RSV-infected and cocultured uninfected cells upregulated LLT1 (top row) compared to uninfected cultures (bottom row). Cell outlines are drawn in white in single-color fluorescent images, and uninfected cells are indicated with arrows in the top row.

To further understand how LLT1 is regulated in respiratory epithelial cells by respiratory-virus infection, cells were infected and 20 h later were costained for RSV and for LLT1. Confocal imaging revealed that LLT1 upregulation occurred in all cells in infected cultures, in both infected and uninfected cells (Fig. 2), but not in wells where all cells were left uninfected. This suggests that soluble factors present in the culture of infected cells were able to mediate the upregulation of LLT1.

### The CD161 ligand LLT1 is rapidly expressed in bronchial epithelial cells in response to TLR ligands.

**FIG 3:**
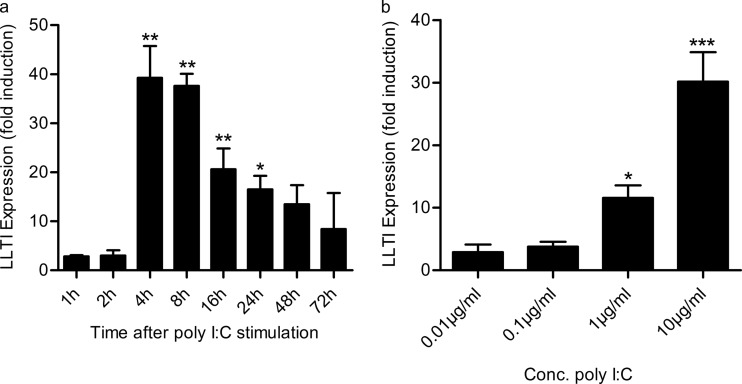
LLT1 expression is rapidly induced on bronchial epithelial cells by poly(I·C). (a) BEAS-2B monolayers were stimulated with 10-μg/ml poly(I·C), and LLT1 gene expression was determined by quantitative PCR at time points up to 72 h. (b) Dose-response curve of LLT1 expression in BEAS-2B cells in response to poly(I·C) stimulation for 4 h. The data are shown as fold induction above unstimulated control wells, normalized to the GAPDH housekeeping gene. The data are means and SD of triplicate wells. *, *P* < 0.05; **, *P* < 0.01; ***, *P* < 0.001 by one-way ANOVA. Conc., concentration.

RSV is detected by pattern recognition receptors, including TLR3 and RIG-I. To determine whether TLR3 ligation can lead to expression of LLT1 and to establish the optimal time point for measurement of LLT1 expression, cells were stimulated with 10 μg/ml poly(I·C) for 1, 2, 4, 8, 16, 24, 48, and 72 h, and mRNA expression was analyzed by quantitative RT-PCR. Expression was found to be induced rapidly and peaked at 4 to 8 h, with an increase of about 35- to 40-fold relative to unstimulated cells, and then declined to 10-fold by 72 h (Fig. 3a). A dose-response analysis of LLT1 expression at 4 h revealed that bronchial epithelial cells were very sensitive to poly(I·C) stimulation and responded over a range of concentrations, reaching statistical significance at 1 and 10 μg/ml (Fig. 3b).

**FIG 4:**
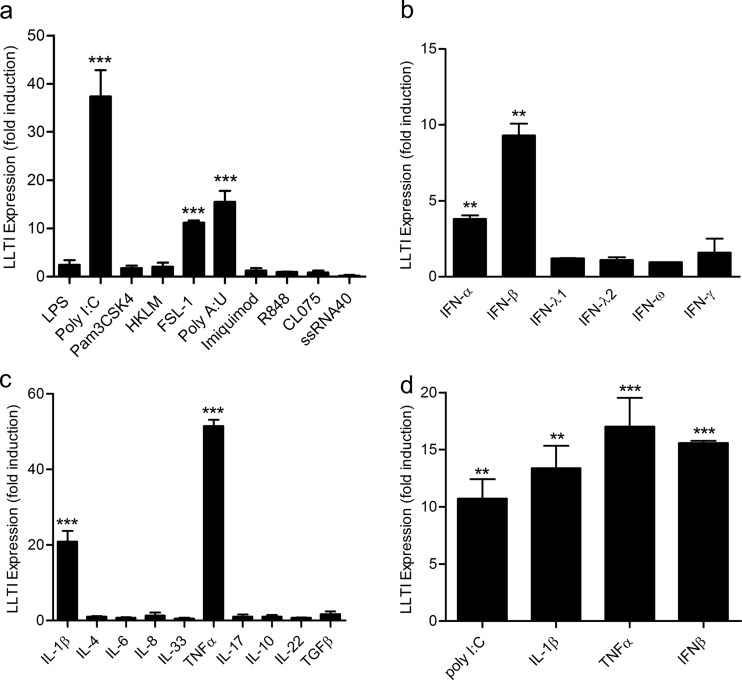
LLT1 is induced by TLR ligands and cytokines. (a to c) BEAS-2B cells were stimulated with TLR ligands (a); type I, type II, and type III interferons (b); or cytokines (c) for 4 h, and the fold induction of LLT1 expression was determined by quantitative PCR. (d) Expression of LLT1 by cultures of primary bronchial epithelial cells from a single donor stimulated for 4 h. The data are shown as fold induction above unstimulated control wells, normalized to the GAPDH housekeeping gene. The data are means and SD of triplicate wells. **, *P* < 0.01, and ***, *P* < 0.001 by one-way ANOVA.

As 4 h was discovered to be the peak of LLT1 expression, BEAS-2B cells were stimulated with various TLR ligands, and expression was assessed by PCR after 4 h. Among the TLR ligands, poly(I·C) (TLR3) revealed the greatest level of LLT1 expression; however, FSL-1 (TLR2/6) also showed a high level of induction (Fig. 4a). The TLR agonists LPS (TLR4), HKLM (TLR2), Pam3CSK4 (TLR2), imiquimod (TLR7), R848 (TLR7/8), CL075 (TLR7/8), ssRNA40/Lyovec (TLR8), and ODN 2006 (TLR9) did not significantly stimulate LLT1 expression at this time point at the concentrations tested. Poly(I·C) can also signal through non-TLR pathways, such as RIG-I. We tested the selective agonist poly(A·U), which signals only through TLR3. This agonist was able to upregulate LLT1, indicating that TLR3 signaling was indeed able to stimulate expression of LLT1 (Fig. 4a). Therefore, LLT1 expression is a specific response to TLR3 ligation, a pathway that is known to be stimulated during respiratory-virus infection (26).

### The CD161 ligand LLT1 is rapidly expressed in bronchial epithelial cells stimulated in response to proinflammatory cytokines.

We then tested cytokines known to be produced during respiratory-virus infections to determine whether they could induce LLT1 expression. Different interferons were tested for the ability to induce LLT1 expression. The type I interferon IFN-β induced a high level of LLT1 expression, as did IFN-α (Fig. 4b). IL-1β (10 ng/ml) and tumor necrosis factor alpha (TNF-α) (10 ng/ml) also induced rapid upregulation of LLT1 transcript in the epithelial cells (Fig. 4c). Other cytokines tested that did not induce a significant change in LLT1 expression at 4 h were IL-4 (10 ng/ml), IL-6 (100 ng/ml), IL-8 (500 ng/ml), IL-33 (50 ng/ml), TGF-β (10 ng/ml), IL-10 (10 ng/ml), IL-17 (10 ng/ml), IL-22 (10 ng/ml), IFN-λ1 (100 ng/ml), IFN-λ2 (100 ng/ml), IFN-ω (1,000 U/ml), and IFN-γ (500 U/ml) (Fig. 4c).

We then wished to confirm that primary human epithelial cells could respond to TLR and cytokine stimuli by upregulating expression of LLT1 in the same way as the BEAS-2B cell line. Cultured normal human bronchial epithelial cells also rapidly expressed LLT1 in response to the cytokines IL-1β, TNF-α, and IFN-β and in response to poly(I·C) (Fig. 4d).

Our data demonstrate that respiratory epithelial cells upregulate expression of the CD161 ligand LLT1 following detection of viral infection via the pattern recognition receptor TLR3 and following stimulation by the proinflammatory cytokines TNF-α, IL-1β, and type I IFNs, which are released early following respiratory-virus infection.

### LLT1 surface expression on respiratory epithelial cells.

**FIG 5:**
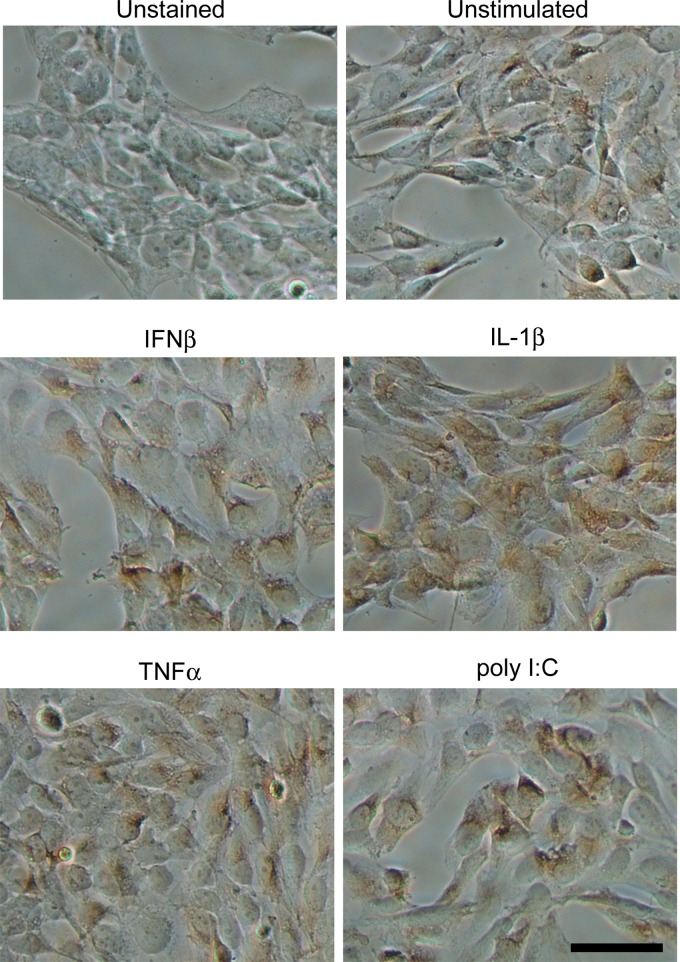
LLT1 protein expression in bronchial epithelial cells. BEAS-2B cells were stimulated with 10,000 U/ml IFN-β, 10 ng/ml IL-1β, 10 ng/ml TNF-α, or 10 μg/ml poly(I·C) for 16 h, and immunohistochemistry was carried out for LLT1 protein expression. LLT1 expression was visualized using DAB substrate, which forms a brown precipitate. Scale bar = 50 μm.

LLT1 is encoded by one of the alternatively spliced transcripts of the *Clec2d* gene located in the natural killer gene complex (27). In order to establish whether the increases in gene expression induced by inflammatory stimuli lead to LLT1 protein on the epithelial cell surface, immunohistochemistry was performed under nonpermeabilizing conditions on BEAS-2B monolayers after 16 h of stimulation. There was some expression of LLT1 in epithelial cells in the absence of stimulation (visualized as brown DAB substrate precipitate) and significant upregulation of expression in cell monolayers stimulated with cytokines or poly(I·C) (Fig. 5). Thus, the changes we observed in LLT1 gene expression led to increased LLT1 protein on the cell surface.

### LLT1 clusters at the immune synapse between respiratory epithelial cells and CD161^+^ T cells.

**FIG 6:**
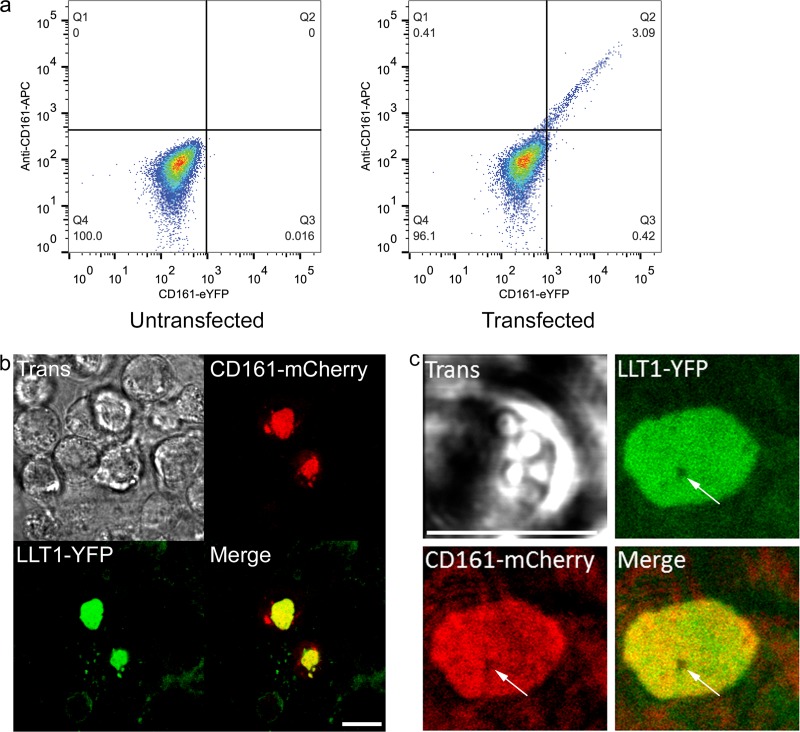
Epithelial LLT1 accumulates at the immune synapse with CD161^+^ T lymphocytes. (a) Flow cytometric analysis of CD161 surface expression as detected using a CD161-APC antibody with or without transfection with a CD161-EYFP construct. (b) BEAS-2B cells were transfected (Trans) to express LLT1-EYFP and were imaged by confocal microscopy interacting with T lymphocytes transfected to express CD161-mCherry. The low-magnification images show many T lymphocytes (small round cells), two of which are transfected with CD161 (red), in contact with BEAS-2B cells transfected with LLT1-EYFP (green). (c) High-magnification image of a single synapse formed with a CD161^+^ lymphocyte showing colocalization of CD161 and LLT1. The arrows indicate a void in the synapse lacking both receptor and ligand. Scale bars = 10 μm.

We wished to determine whether LLT1 expressed on epithelial cells could indeed interact with lymphocytes expressing CD161 and to understand the nature of the interaction. We utilized the Jurkat T cell line and used a CD161 antibody to confirm that these T cells do not normally express CD161 on the cell surface but do so when transiently transfected with a CD161-EYFP construct (Fig. 6a).

Confocal imaging of LLT1-EYFP-transfected epithelial cells interacting with transiently transfected T cells expressing CD161-mCherry was performed. A low proportion of T cells were transfected, allowing comparison between CD161^+^ (red) and CD161^−^ lymphocytes (seen as round cells in Fig. 6b). When CD161^+^ lymphocytes (seen as red cells in Fig. 6b) came into contact with the LLT1-expressing bronchial epithelial cells (green) (Fig. 6b), both molecules accumulated at the site of contact. There was no accumulation of LLT1 at sites of contact with the majority untransfected T cells (visible in the transmission image, but not in the red or green channels) (Fig. 6b). At the site of interaction, the distribution of LLT1-EYFP and CD161-mCherry clearly overlapped, demonstrating coclustering of these molecules and the requirement for lymphocyte CD161 for LLT1 clustering (Fig. 6b; shown at high magnification in Fig. 6c). Of 40 synapses observed in transfected T cells, 39 (97.5%) showed prominent coclustering of LLT1 and CD161. Of 159 untransfected Jurkat cells, none (0%) led to prominent clustering of LLT1 at the T cell-epithelial cell interface. The large, synapse-wide accumulations of CD161/LLT1 were initially formed as small dynamic clusters at the T cell-bronchial epithelial cell interface, which coalesced (not shown). These structures were further characterized by receptorless “voids,” which perforated otherwise homogeneous accumulations of LLT1/CD161 (Fig. 6c). This suggests that upon upregulation of LLT1 on epithelial cells, as is seen following respiratory-virus infection, the ligand can interact with CD161 and accumulate at the immune synapse formed with CD161-expressing lymphocytes.

## DISCUSSION

Understanding the mechanisms by which respiratory-virus infection promotes the activation of lymphocytes in the lung is a key goal. The importance of the epithelium as a regulator of leukocyte responses in the lung is becoming increasingly apparent. Here, we show that the CD161 ligand LLT1 is expressed on bronchial epithelial cells in response to RSV infection and TLR ligation and in response to the proinflammatory cytokines type I interferons, IL-1β, and TNF-α. We demonstrate that LLT1 expressed in the respiratory epithelium traffics to the epithelial surface and interacts at the immune synapse with CD161^+^ lymphocytes. The CD161 receptor is expressed on proinflammatory lymphocytes at mucosal sites, including the lungs, and thus, expression of LLT1 on respiratory epithelial cells following viral infection links innate and adaptive immunity and could be an important regulator of lymphocyte activation in the lung.

LLT1 transcript expression in response to TLR3 recognition of poly(I·C) peaked at 4 to 8 h. This is remarkably rapid compared to previously published observations of other cell types. LLT1 expression on T and B lymphocytes and dendritic cells was reported after 24 to 72 h of stimulation (8, 10). In whole-blood peripheral blood mononuclear cells (PBMC), ligands for TLR3, TLR4, TLR7, TLR8, or TLR9 were able to induce LLT1 expression (8). In dendritic cells, ligands for TLR3, TLR4, TLR 7/8, and TLR9 induce LLT1, and in B cells, TLR7/8 and TLR9 induce expression (8, 10). This is in contrast to our findings of a lack of induction by the TLR4 ligand LPS, the TLR9 ligand CpG, or TLR7/8 ligands in the epithelium. We did find expression induced by FSL-1, a synthetic mycobacterium-derived mycoprotein that stimulates TLR 2/6. Interestingly, mycobacteria can also increase expression of the MR1 molecule, which presents antigen to MAIT cells (28, 29). MAIT cells are abundant, unconventional, antimicrobial CD8^+^ CD161^+^ T cells found at mucosal surfaces that may play roles in respiratory-bacterial infection (30–32). Therefore, LLT1 is induced in a number of cell types in response to microbial stimuli and thus provides a link between pattern recognition and lymphocyte activation.

Detection of viral infection by the host leads to the production of type I interferons. We show that IFN-β and IFN-α are potent inducers of LLT1 expression in epithelial cells. In cocultures of RSV-infected and uninfected cells, both infected and uninfected cells upregulated LLT1 expression, indicating that soluble mediators could induce LLT1 expression. Interestingly, staining appeared brighter in uninfected cells. This could be because of specific or nonspecific inhibition of LLT1 synthesis in infected cells. Alternatively, it is consistent with inhibition of mediator signaling in infected cells, particularly RSV nonstructural protein (NS1 and NS2) inhibition of type I interferon signaling. We also observed induction of expression by the proinflammatory cytokines TNF-α and IL-1β. It will be interesting to find out whether these cytokines can induce LLT1 expression on other cell types and whether they in fact indirectly mediate the effects of other TLR ligands or virus infections previously reported by others.

From our data, it can be concluded that cell surface LLT1 interacts to form an immune synapse with lymphocytes expressing the receptor CD161. CD161 has been shown to be involved in a number of aspects of the lymphocyte response. CD161 can inhibit cytotoxicity and IFN-γ secretion of NK cells (7, 25). In T cells, the role of CD161 continues to be debatable. Some authors report no effects or inhibitory effects on T cells (8); however, others demonstrate costimulation of conventional and CD1d-restricted T cells and thymocytes following CD161 ligation, which promotes their activation, proliferation, and cytokine secretion (5–7, 9, 10).

CD161^+^ lymphocytes are important proinflammatory lymphocytes in respiratory disease. Taken together, our data demonstrate that LLT1 is expressed on the epithelial surface in response to TLR ligands and by proinflammatory cytokines that would be released during respiratory-virus infection. This suggests a mechanism by which activation of CD161^+^ lymphocytes may be regulated by the epithelium during infection. As a consequence, NK cell activation may be reduced, which may protect the epithelial cells from NK-mediated lysis. Conversely, activation of proinflammatory CD161^+^ lymphocytes, particularly Th17 and Tc17 subsets, may be promoted. LLT1 expression thus provides a link between respiratory-virus infection and activation of proinflammatory lymphocytes. As such, LLT1 may represent a novel target for regulation of excessive respiratory inflammation caused by acute infection, such as in RSV bronchiolitis, and in viral exacerbations of chronic diseases, such as chronic obstructive pulmonary disease (COPD) and asthma.
